# Mesenchymal stem cells exert their anti-asthmatic effects through macrophage modulation in a murine chronic asthma model

**DOI:** 10.1038/s41598-022-14027-x

**Published:** 2022-06-13

**Authors:** Ruth Lee Kim, Ji-Young Bang, Jeonghyeon Kim, Yosep Mo, Yujin Kim, Chun-Geun Lee, Jack A. Elias, Hye Young Kim, Hye-Ryun Kang

**Affiliations:** 1 Institute of Allergy and Clinical Immunology, Seoul National University Medical Research Center, Seoul National University College of Medicine, Seoul, Korea; 2 Department of Translational Medicine, Seoul National University College of Medicine, Seoul, Korea; 3 Department of Molecular Microbiology and Immunology, Brown University, Providence, Rhode Island USA; 4 Department of Internal Medicine, Seoul National University College of Medicine, Seoul, Korea; 5 Laboratory of Mucosal Immunology in Department of Biomedical Sciences, Seoul National University College of Medicine, Seoul, Korea

**Keywords:** Innate immunity, Monocytes and macrophages, Immunology, Interleukins, Asthma, Stem-cell therapies, Mesenchymal stem cells

## Abstract

Despite numerous previous studies, the full action mechanism of the pathogenesis of asthma remains undiscovered, and the need for further investigation is increasing in order to identify more effective target molecules. Recent attempts to develop more efficacious treatments for asthma have incorporated mesenchymal stem cell (MSC)-based cell therapies. This study aimed to evaluate the anti-asthmatic effects of MSCs primed with Liproxstatin-1, a potent ferroptosis inhibitor. In addition, we sought to examine the changes within macrophage populations and their characteristics in asthmatic conditions. Seven-week-old transgenic mice, constitutively overexpressing lung-specific interleukin (IL)-13, were used to simulate chronic asthma. Human umbilical cord-derived MSCs (hUC-MSCs) primed with Liproxstatin-1 were intratracheally administered four days prior to sampling. IL-13 transgenic mice demonstrated phenotypes of chronic asthma, including severe inflammation, goblet cell hyperplasia, and subepithelial fibrosis. Ly6C^+^M2 macrophages, found within the pro-inflammatory CD11c^+^CD11b^+^ macrophages, were upregulated and showed a strong correlation with lung eosinophil counts. Liproxstatin-1-primed hUC-MSCs showed enhanced ability to downregulate the activation of T helper type 2 cells compared to naïve MSCs in vitro and reduced airway inflammation, particularly Ly6C^+^M2 macrophages population, and fibrosis in vivo. In conclusion, intratracheal administration is an effective method of MSC delivery, and macrophages hold great potential as an additional therapeutic target for asthma.

## Introduction

Asthma, characterized by airway hyperresponsiveness, shortness of breath, and excessive mucus production, is a chronic airway inflammatory disease that shows an increasing prevalence rate every year^1^. Advanced research in drug development and drug delivery has led to improvements in asthma treatment for the past 50 years. However, currently available drugs still face issues of limited efficacy, sustained morbidity, and notable adverse effects^2^.

Recent attempts to develop a more efficacious treatment for asthma have incorporated mesenchymal stem cells (MSCs), multipotent stromal cells that have extensive abilities to differentiate into a variety of cell types. Their cross-species immunosuppressive activities and reparative immunoregulatory properties make them an attractive therapeutic agent for asthma^3,4^, and many studies have successfully demonstrated the anti-inflammatory and anti-asthmatic effects of MSCs with various origins, dosages, and frequencies. Early studies, however, were limited to injecting the MSCs intravenously^5,6^. This method, however, possesses a disadvantage in delivery efficiency, as a large number of cells are lost before reaching the lung^7^. To address this limitation, an intratracheal administration method was introduced, targeted to improve the delivery efficacy and augment the therapeutic effects of the MSCs.

Another method that attempts to enhance the therapeutic efficacy of MSCs is priming, exposing the MSCs to diverse molecules prior to administration. MSCs with high glutathione (GSH) have an improved stem cell function and showed an enhanced therapeutic effect in asthma^8,9^. Liproxstatin-1, a known ferroptosis inhibitor, can enhance stem cell function by effectively suppressing lipid peroxidation^10^. Therefore, we hypothesized that Liproxstatin-1 could improve the therapeutic effect of stem cells on asthma by reducing reactive oxygen species mediated damage in the membrane lipid of MSC.

Although the underlying mechanisms leading to the pathology of asthma are still unclear, it has long been considered a T helper type 2 (T_H_2)-dominant disease^11,12^. Interleukin (IL)-13, one of the most predominant T_H_2 cytokines, is known to play pivotal roles in exhibiting asthmatic phenotypes, including, but not limited to, eosinophilia in the respiratory organs, mucus hypersecretion, and airway remodeling. Consistent with findings in animal models, it has been clinically proven that asthma patients showed elevated levels of IL-13 in the blood and sputum^13–16^. Reduced IL-13 secretion from T_H_2 cells, however, does not always lead to an alleviation of asthma. Here, we hypothesized that there exists another cell population that induces T_H_2-like responses and contributes significantly to the pathogenesis of asthma. M2 macrophages, characterized by their expression of *Mrc1*, *Arg1*, and *Chil3* are well-qualified candidates as they secrete IL-13, are known to activate T cells^13,17,18^ and are upregulated in the blood of asthma patients^19,20^.

Using a murine chronic asthma model with genetically modified transgenic (TG) mice that constitutively overexpress IL-13 in the lung, the study aimed to evaluate the anti-asthmatic effects of human umbilical cord-derived MSCs (hUC-MSCs) primed with Liproxstatin-1. Additionally, we sought to further evaluate the changes within macrophage populations and their characteristics in asthmatic conditions.

## Results

### Liproxstatin-1-primed hUC-MSCs reduced airway inflammation and fibrosis in a murine chronic asthma model

In this study, we used Liproxstatin-1-primed MSCs since Liproxstatin-1-primed MSCs have been shown to exhibit enhanced overall potential anti-asthmatic capacities compared to naïve MSCs, and their therapeutic effects were retained in xenogeneic recipients. (Figs. S1, S2).

**Figure 1:**
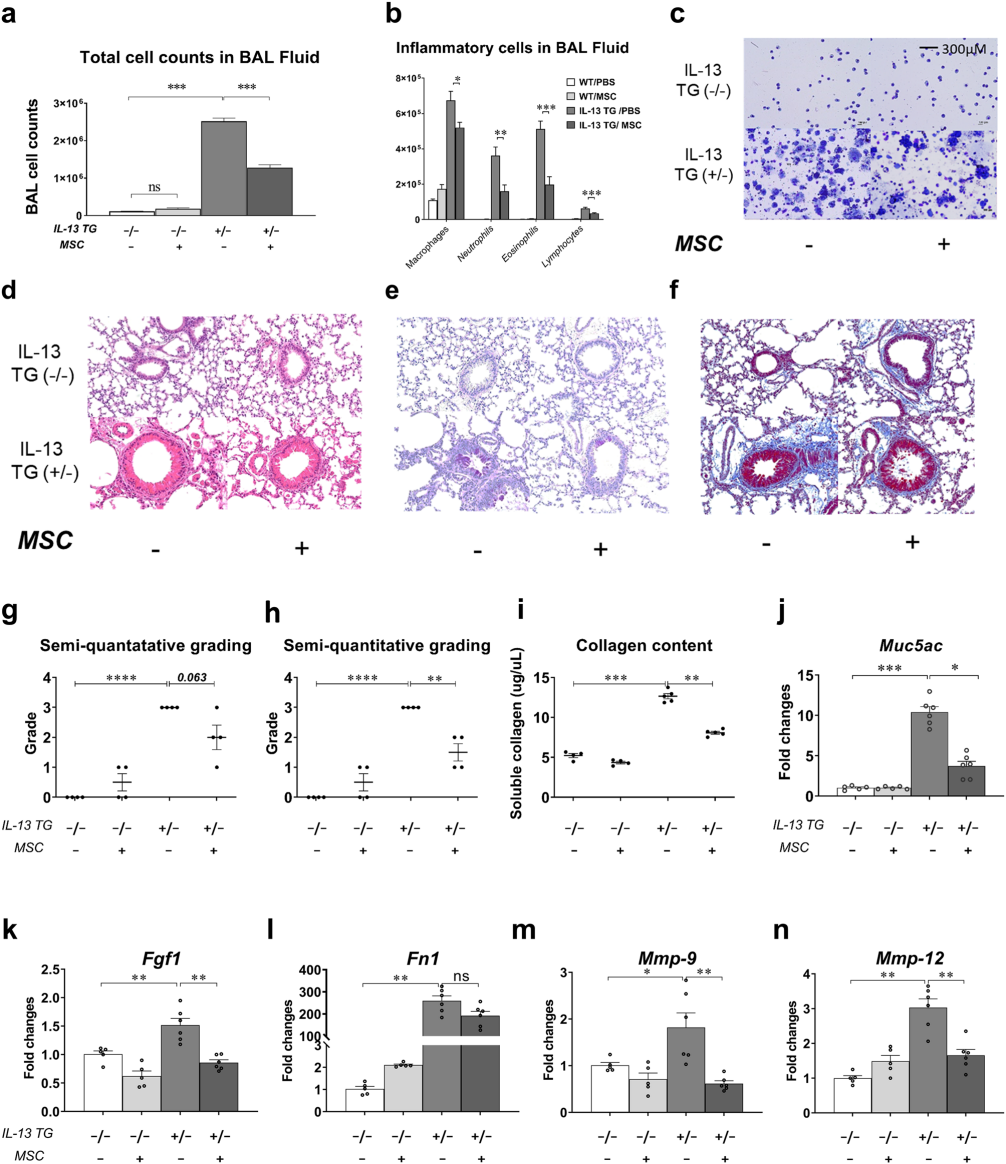
Liproxstatin-1-primed hUC-MSCs alleviated airway inflammation mitigated pulmonary fibrosis in vivo*.* (**a**) Shows the result of total cell counts and (**b**) shows differential counts of macrophages, neutrophils, eosinophils, and lymphocytes in BAL fluid. (**c**) Shows microscopic images of Diff-Quick-stained slides of cytocentrifuged BAL fluid (× 40). Samples from IL-13 TG groups were diluted with doubled amount of PBS compared with the samples from the wild type groups. (**d**–**e**) shows histological comparison between the groups at a power of 20. H&E (**d**) and PAS (**e**) stains were used. (**f**) Shows Masson’s Trichrome-stained slides of each group at low power (× 20). (**g**–**h**) shows semi-quantitative grading results of H&E staining (**g**) and PAS staining (**h**). Sircol collagen assay was performed to quantify the soluble collagen contents in the homogenates of the lungs (**i**). (**j**–**n**) represents relative gene expressions of *Muc5ac (j)*, *Fgf1 (k)*, *Fn1 (l)*, *Mmp-9 (m)*, and *Mmp-12 (n)* compared to the housekeeping, *Gapdh.* Each value in these panels is from a different individual and the mean ± SEM are illustrated. All results are representative of at least three independent experiments. *p < 0.05, **p < 0.01, ***p < 0.001, ****p < 0.0001, *ns* not significant. (by Kruskal–Wallis test using GraphPad Prism 7, https://www.graphpad.com).

The anti-asthmatic abilities of Liproxstatin-1-primed hUC-MSCs were further assessed using a murine model of chronic asthma. Compared to the healthy control group, the disease control group showed a significant increase in total inflammatory cell counts with a rise in the numbers of macrophages, neutrophils, and eosinophils in the bronchoalveolar lavage (BAL) fluid (Fig. 1A–C). No significant difference was observed between the two Wild type (WT) mice groups. Among the two disease groups, the MSC-treated IL-13 mice showed a significant decrease in the numbers of all analyzed cell types, especially eosinophils.

In accordance with the BAL fluid differential count data, histological analysis with Hematoxylin/eosin (H&E) staining displayed slight decrease of inflammatory cell recruitment around the vessels and airways in the MSC-treated disease group, compared to the disease control group (Fig. 1D,G). The quasi-absence of goblet cell hyperplasia (Fig. 1E,H) and the reduced gene expression of *Muc5ac*, a gene linked to mucus secretion, in the MSC-treated IL-13 TG mice (Fig. 1J) also indicated that Liproxstatin-1-primed hUC-MSCs exert anti-inflammatory effects in a murine model of chronic asthma.

Masson's trichrome (MT)-stained slides revealed excessive collagen deposition in the airway of the disease control group. In the MSC-treated disease group, on the other hand, a significant reduction of the collagen fibers was observed (Fig. 1F), and the Sircol assay confirmed that approximately 30% of soluble collagen was reduced in the disease group upon MSC injection (Fig. 1I). A remarkable downregulation in the mRNA levels of airway remodeling-related genes, such as fibrosis growth factor-1 (*Fgf-1*), fibronectin-1 (*Fn-1*), matrix metallopeptidase-9 (*Mmp-9*), and *Mmp-12,* in the MSC-treated IL-13 TG mice confirmed that Liproxstatin-1-primed hUC-MSCs not only mitigated inflammation, but also abated airway remodeling at the genetic level (Fig. 1K–N).

### Liproxstatin-1-primed hUC-MSCs led to alterations in lung macrophage populations

**Figure 2:**
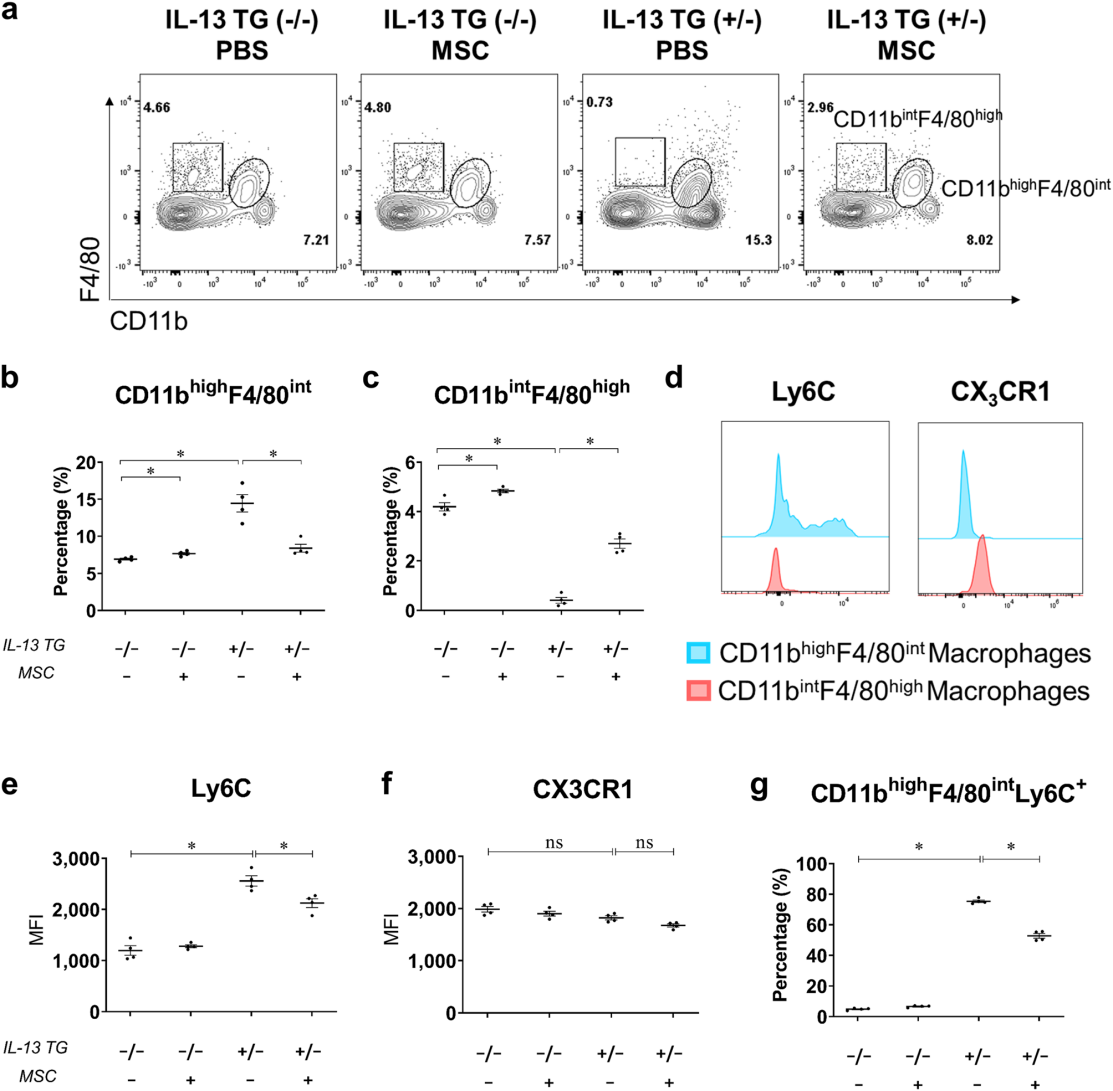
Liproxstatin-1-primed hUC-MSCs altered macrophage populations in the lung. (**a**) Flow cytometry data showing two distinct macrophage populations that differ by levels of CD11b and F4/80 expression. Numbers represent percentages among total CD45^+^ leukocytes, excluding SiglecF^+^CD11c^−^ eosinophils. Based on the flow cytometry representations shown in (**a**), percentages of CD11b^high^F4/80^int^ macrophages (**b**) and CD11b^int^F4/80^high^ macrophages (**c**) are each portrayed in graphs. (**d**) Represents a histogram of Ly6C and CX_3_CR1 expression levels of CD11b^int^F4/80^high^ macrophages and CD11b^high^F4/80^int^ macrophages. (**e**) Represents the MFI of Ly6C of CD11b^high^F4/80^int^ macrophages and (**f**) shows the MFI of CX_3_CR1 of CD11b^int^F4/80^high^ macrophages. (**g**) Shows the percentages of Ly6^+^ macrophages among CD11b^high^F4/80^int^ macrophages. Flow cytometry data were analyzed by the FlowJo Software v10.6, https://www.flowjo.com. Each value in these panels is from a different individual and the mean ± SEM are illustrated. All results are representative of at least three independent experiments. *p < 0.05, *ns* not significant. (by Kruskal–Wallis test using GraphPad Prism 7, https://www.graphpad.com).

The most evident differences between the WT and IL-13 TG mice were observed within the macrophage populations. The disease control group showed diminished CD11b^int^F4/80^high^ macrophage counts and a nearly doubled number of CD11b^high^F4/80^int^ macrophages compared to those of the wild-type groups (Fig. 2A–C). Upon administration of Liproxstatin-1-primed hUC-MSCs, partial recovery of the dissipated CD11b^int^F4/80^high^ macrophage population and downregulation of CD11b^high^F4/80^int^ macrophages were observed in the IL-13 TG mice, indicating that Liproxstatin-1-primed hUC-MSCs caused alterations in the macrophage populations in the lung.

To further characterize the CD11b^int^F4/80^high^ macrophages and the CD11b^high^F4/80^int^ macrophages, the expression levels of Ly6C and CX_3_CR1 were analyzed, in order to distinguish between different macrophage populations^21^. All CD11b^int^F4/80^high^ macrophages from the healthy control group expressed low levels of Ly6C and high levels of CX_3_CR1. On the other hand, CD11b^high^F4/80^int^ macrophages were divided into Ly6C^+^ and Ly6C^−^ populations and their CX_3_CR1 expression levels were low regardless of their Ly6C expression (Fig. 2D). The mean fluorescence intensity (MFI) of CD11b^high^F4/80^int^Ly6C^+^ macrophages, as well as the percentages of CD11b^high^F4/80^int^Ly6C^+^ macrophages, were upregulated in the disease control group, and that of CD11b^int^F4/80^high^CX_3_CR1^+^ macrophages remained constant in all groups. Both the MFI and the percentages of CD11b^high^F4/80^int^Ly6C^+^ macrophages showed a significant reduction upon intratracheal administration of hUC-MSCs (Fig. 2E–G).

### CD11c^+^CD11b^+^ macrophages are upregulated in asthmatic conditions

**Figure 3:**
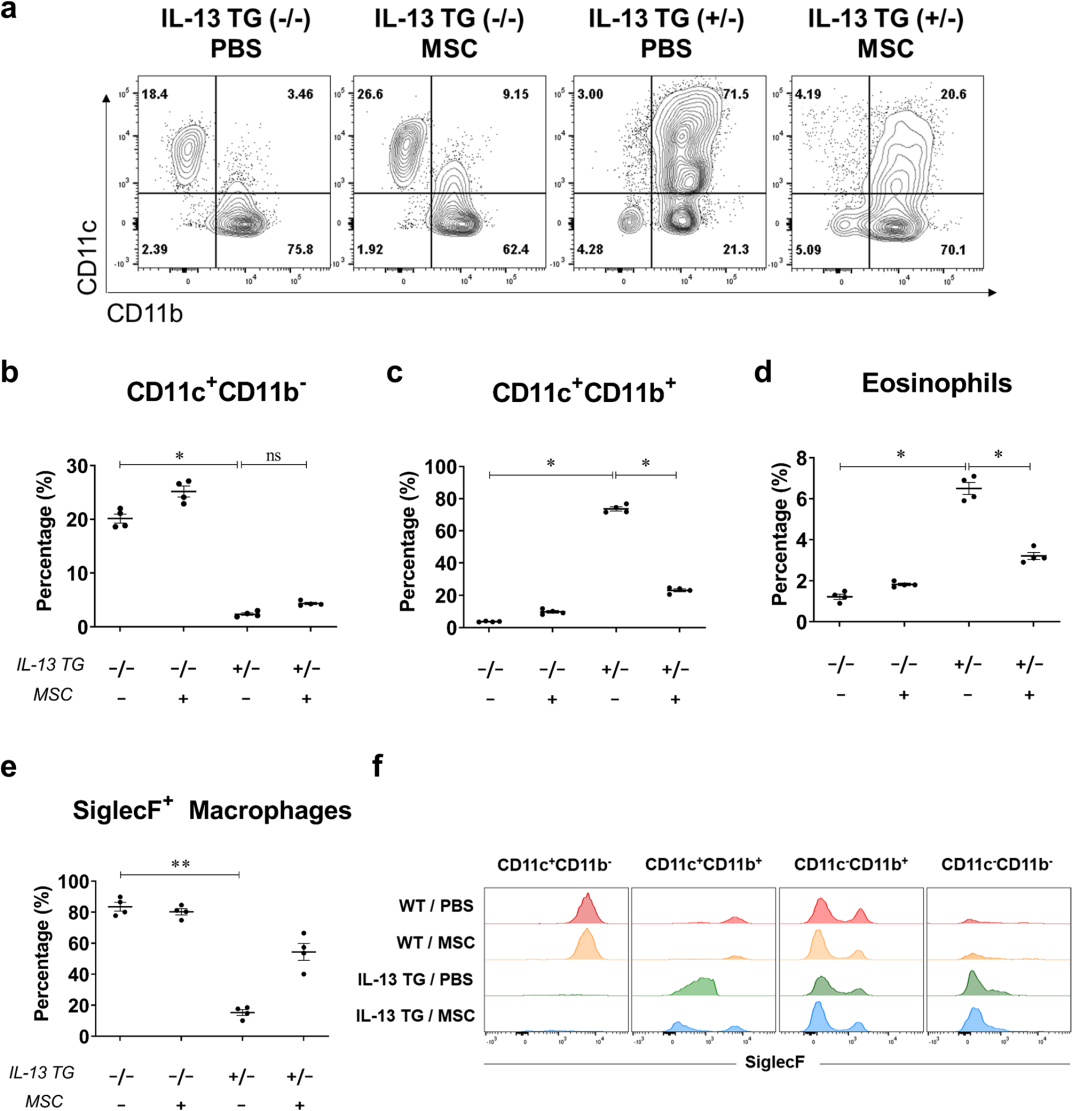
Liproxstatin-1-primed hUC-MSCs caused a reduction in the number of CD11c^+^CD11b^+^ pro-inflammatory macrophages. (**a**) shows a flow cytometric representation of macrophage populations divided into four groups by their expression of CD11b and CD11c. (**b**, **c**) show percentages of CD11c^+^CD11b^−^ (**b**) and CD11c^+^CD11b^+^ macrophages (**c**) among total macrophages. (**d**) Percentage of lung eosinophils out of total CD45^+^ cells are represented in (**d**). (**e**) shows the absolute counts of CD11c^+^CD11b^+^SiglecF^+^ macrophages and (**f**) represents a histogram of SiglecF expressions of CD11c^+^CD11b^+^, CD11c^**−**^CD11b^+^, CD11c^+^CD11b^−^, and CD11c^−^CD11b^−^ macrophages. Flow cytometry data were analyzed by the FlowJo Software v10.6, https://www.flowjo.com. Each value in these panels is from a different individual and the mean ± SEM are illustrated. All results are representative of at least three independent experiments. *p < 0.05, **p < 0.01, ns, not significant. (by Kruskal–Wallis test using GraphPad Prism 7, https://www.graphpad.com).

CD11b^int^ to CD11b^high^ macrophages were then subdivided based on CD11c expression*.* In the wild-type mice groups, the CD11c^+^CD11b^−^ macrophages constituted approximately 20%–25% of the total macrophages, in contrast to the two disease groups where they constituted less than 5% of the population (Fig. 3A,B). This population has a correspondence with SiglecF^+^CD11c^+^ macrophage population which is regarded as resident alveolar macrophages (Fig. S3). The majority of the macrophages in the disease groups were found to express both CD11c and CD11b, which showed a remarkable decrease after MSC injection (Fig. 3A,C). In accordance with the CD11c^+^CD11b^+^ macrophages, lung eosinophils counts were elevated in IL-13 TG mice and were downregulated upon MSC treatment (Fig. 3D). When the CD11c^+^CD11b^+^ macrophages were further characterized by their SiglecF expression, that of both wild-type groups showed high SiglecF expression, whereas those of the disease control group showed low expression. In the disease group injected with Liproxstatin-1-primed hUC MSCs, a co-existence of SiglecF^+^ and SiglecF^-^ macrophages was discovered (Fig. 3E,F). Although the observation was not statistically significant, the phenomenon was clearly represented by two distinct peaks in the histograms produced by flow cytometry. The conspicuous difference in SiglecF expression between the WT and IL-13 TG mice was not seen in the rest of the populations.

### Ly6C^+^M2 macrophages among CD11c^+^CD11b^+^ macrophages are potential markers for asthma

**Figure 4:**
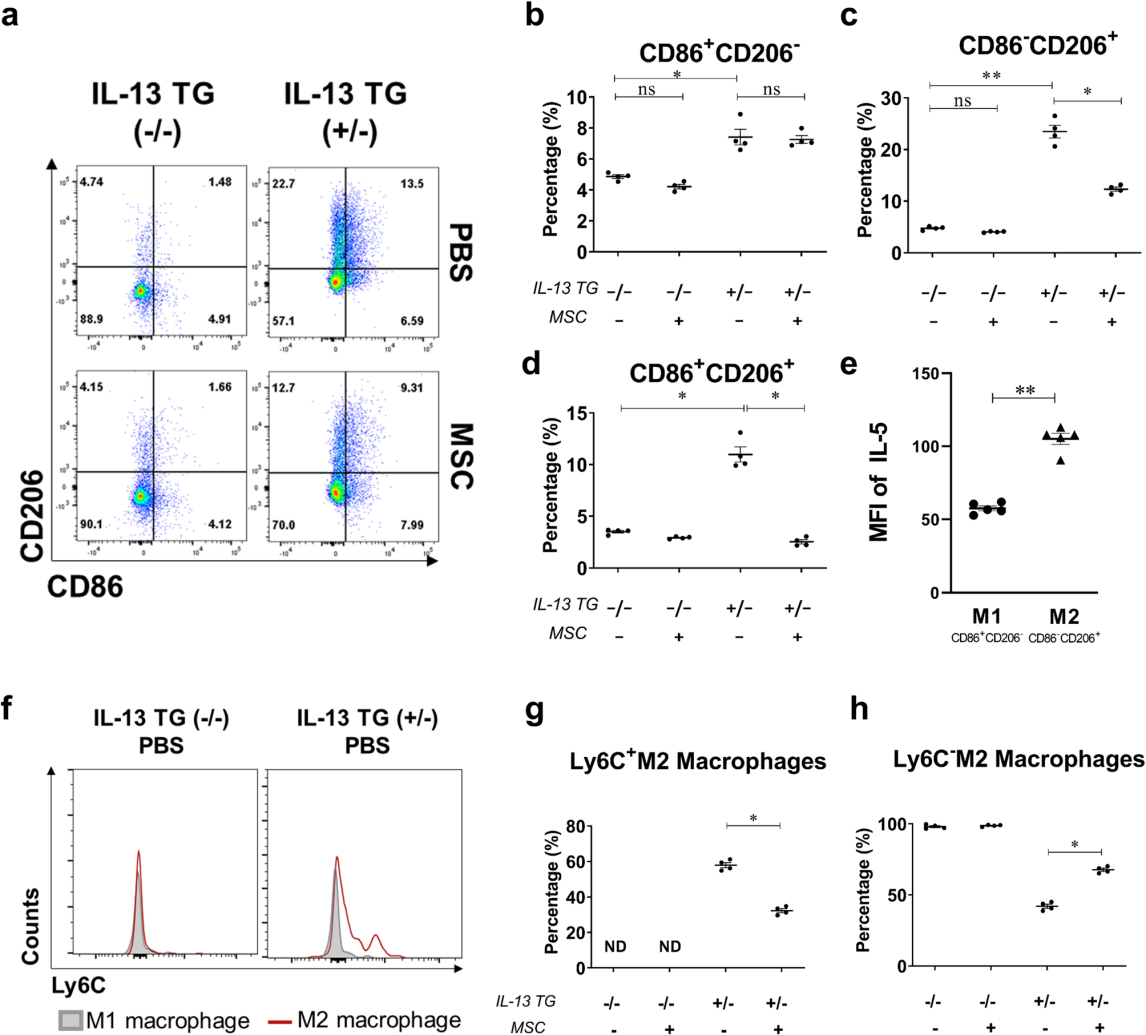
Ly6C^+^M2 macrophages are key mediators of asthmatic phenotypes. (**a**) Flow cytometric data on M1 and M2 macrophages of CD11c^+^CD11b^+^ macrophages. The percentages of CD86^+^CD206^−^ M1 (**b**), CD86^−^CD206^+^ M2 macrophages (**c**), and CD86^+^CD206^+^ macrophages (**d**) are represented in scatter plots. (**e**) the MFI of IL-5 in CD86^+^CD206^−^ M1 and CD86^−^CD206^+^ M2 macrophages in IL-13 TG mice. (**f**) Shows Ly6C expression levels in M1 and M2 macrophages of WT and IL-13 TG mice. (**g**, **h**) shows the ratio of Ly6C^+^M2 and LY6C^−^M2 macrophage populations among the CD11c^+^CD11b^+^ macrophages. Flow cytometry data were analyzed by the FlowJo Software v10.6, https://www.flowjo.com. Each value in these panels is from a different individual and the mean ± SEM are illustrated. All results are representative of at least three independent experiments. *p < 0.05, **p < 0.01, *ns* not significant. (by Kruskal–Wallis test using GraphPad Prism 7, https://www.graphpad.com).

Next, the polarization status of CD11c^+^CD11b^+^ macrophages was assessed. To avoid a possible misleading interpretation, the F4/80^+^ CD11c^+^CD11b^−^ alveolar macrophages were excluded, as they express surface markers for both M1 and M2 macrophages. CD86^+^CD206^−^ M1, CD86^−^CD206^+^ M2, and CD86^+^CD206^+^ types of CD11c^+^CD11b^+^ macrophages were upregulated in the disease control group. IL-13 TG mice that received hUC-MSCs showed reductions in M2 macrophages and CD86^+^CD206^+^ populations (Fig. 4A–D). M2 macrophages expressed more IL-5 than M1 macrophages (Fig. 4E). It was found that neither M1 nor M2 macrophages from the healthy WT groups showed upregulated Ly6C expression. The disease groups, on the other hand, showed inflated Ly6C expression levels in the M2 macrophages, while showing no changes within the M1 macrophages (Fig. 4F). These results suggested that M2 macrophages adopt Ly6C expression under IL-13 enriched conditions. It was also found that the ratio of the Ly6C^+^M2 macrophages was significantly lower in the disease group treated with MSC injection than in the disease group without MSC injection (Fig. 4G–H), implying that the Ly6C^+^M2 macrophages may be highly associated with the pathogenesis of asthma and the therapeutic mechanism of hUC-MSCs.

### The anti-asthmatic effects of liproxstatin-1-primed hUC-MSCs were carried out by macrophages

**Figure 5:**
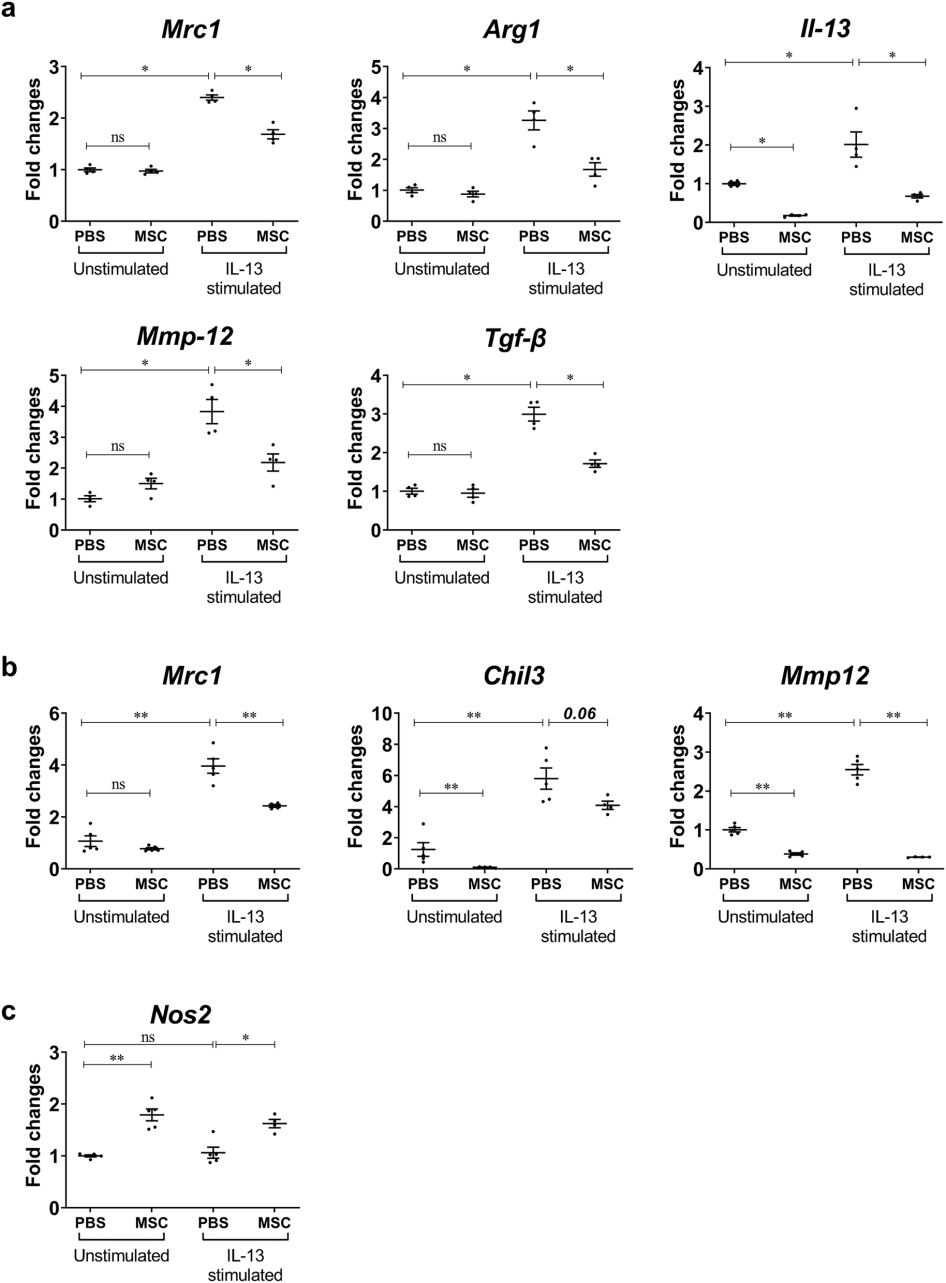
Effects of Liproxstatin-1-primed hUC-MSCs on murine macrophages. (**a**–**c**) Murine macrophages were co-cultured with hUC-MSCs in a trans-well system and ex vivo alveolar macrophages (**a**) and bone marrow-derived macrophages (**b**, **c**) were subjected to RT-qPCR analysis for mRNA evaluation of markers of polarized macrophages. *Gapdh* and *Hprt1* were used as internal controls. Values in all the panels are mean ± SEM. All results are representative of at least three independent experiments. *p < 0.05, **p < 0.01, *ns* stands for not significantly different (by Kruskal Wallis test using GraphPad Prism 7, https://www.graphpad.com). *Il-13* interleukin 13, *Mmp-12* matrix metallopeptidase 12, *Tgf-β* transforming growth factor beta*.*

To confirm the direct effects of hUC-MSCs on macrophage polarization, ex vivo alveolar macrophages (Fig. 5A) and bone marrow-derived macrophages (Fig. 5B,C) obtained from WT C57BL/6 mice were stimulated with IL-13 and co-cultured with/without Liproxstatin-1-primed hUC-MSCs. The data suggested that MSCs can work solely with M2 macrophages to reduce expression of genes related to M2 polarization and airway remodeling, which together mitigate chronic asthma (Fig. 5A,B). Whereas, expression of *Nos2*, M1 macrophages related gene in bone marrow-derived macrophages, was upregulated by MSC (Fig. 5C).

## Discussion

In this study, we evaluated the therapeutic abilities of Liproxstatin-1-primed hUC-MSCs against chronic asthmatic conditions and elucidated their effects on the modulation of macrophage phenotypes.

Starting from an in vitro experiment with human peripheral blood mononuclear cell (PBMC), this study proved that Liproxstatin-1-primed MSCs, compared to naïve MSCs, exerted greater abilities to inhibit activation of T_H_2 and T_H_17 cells and to promote differentiation into Tregs. As T_H_2 and T_H_17 cells are known to significantly contribute to asthmatic phenotypes^22–26^, it was proven that Liproxstatin-1-primed hUC-MSCs hold a greater therapeutic potential for treating asthma than do naïve MSCs.

Through in vivo studies with IL-13 TG mice displaying a majority of the features seen in chronic asthma patients, we confirmed the intratracheal delivery method retained anti-asthmatic abilities, while ensuring the absence of xenogeneic effects, as already proven by previous studies^27,28^. Intratracheal injection of Liproxstatin-1-primed hUC-MSCs into IL-13 TG mice resulted in a remarkable decrease in eosinophil and other immune cell infiltrations into the respiratory tissues. Reduced mucus production and assuaged airway remodeling further substantiated the ability of Liproxstatin-1-primed hUC-MSCs to mitigate asthma in vivo.

We also corroborated that macrophages play key roles in the pathogenesis of asthma. The constitutive IL-13 overexpressing mice showed no signs of activation or proliferation of T cells and innate lymphoid cells (ILC) (data not shown), indicating that the observed anti-asthmatic and anti-fibrotic effects of Liproxstatin-1-primed hUC-MSCs were not mainly manifested through changes in T cells or ILCs, as is commonly acknowledged in allergen asthma models. Here, we presumed that there must be other cell types that are activated by excess IL-13. Macrophages are one of the top candidates, as their polarization status is known to be altered under asthmatic conditions and they are also highly associated with T_H_2 cells^18^. We hypothesized that the changes in macrophage modulation could partially explain the anti-asthmatic responses of Liproxstatin-1-primed hUC-MSCs that cannot be explained by T_H_2 cells or ILCs.

A pronounced difference in macrophages was noted between the healthy and disease groups, as we hypothesized. The CD11b^high^F4/80^int^ macrophages, which were nearly doubled in the IL-13 TG mice, were further divided into two distinct populations: Ly6C^high^ monocyte-derived pro-inflammatory macrophages and homeostatic Ly6C^low^ interstitial macrophages^29,30^. The CD11b^high^F4/80^int^Ly6C^high^ macrophages showed low levels of CX_3_CR1, which corresponded to the phenotypes of pro-inflammatory classical monocytes that differentiate into macrophages upon detecting extravasation of pro-inflammatory cytokines^31,32^. The population in the disease control group showed that CD11b^high^F4/80^int^Ly6C^high^ macrophages may reflect, at least to some extent, the severity of asthma. The IL-13 TG mice also showed an increased population of CD11c^+^CD11b^+^ macrophages, which are pro-inflammatory and secrete IL-6, IL-12, and tumor necrosis factor (TNF)-α^33,34^. Their strong correlation with lung eosinophils suggests that this population may be in charge of crosstalk with T_H_2 cells, which leads to asthmatic phenotypes.

SiglecF is a marker specific for murine lung-resident alveolar macrophages that is not expressed by interstitial or inflammatory macrophages^35,36^. Eosinophils also express SiglecF but can be differentiated by their lack of CD11c expression differently from macrophages. Although the function of SiglecF on macrophages is not fully understood, it is often used as a marker for immunoregulation in inflammatory environments since SiglecF-expressing alveolar macrophages are mainly associated with maintaining homeostasis and their absence aggravate lung inflammation^37,38^. The homeostatic role of alveolar macrophages is well known by macrophage depletion experiments with clodronate, which resulted in the increase of eosinophils and the concentration of Th2 cytokines in BAL fluid^39^. In addition, the adoptive transfer of alveolar macrophages abrogated bronchial hyperresponsiveness^40^. In our experiment, CD11c^+^CD11b^-^ macrophages, the main population expressing SiglecF in wild type control, were depleted in IL-13 TG mice. In addition, CD11c^+^CD11b^+^ macrophages also expressed SiglecF in a lesser degree in wild type control, which was abrogated in IL-13 TG mice but restored by intratracheal MSCs. Therefore, the recovery of SiglecF^+^ macrophages by MSCs in IL-13 TG mice might contribute to anti-asthmatic effect in part by replenishing homeostatic alveolar macrophages.

The polarization status of the CD11c^+^CD11b^+^ macrophages revealed that Liproxstatin-1-primed hUC-MSCs resulted in a reduction of both M1 and M2 CD11c^+^CD11b^+^ macrophage population sizes. Additionally, CD86^+^CD206^+^ populations showed the same trends in both M1 and M2 macrophages: upregulated in the disease control group and downregulated upon MSC injection. This population was inferred to be alveolar macrophage-derived populations as they express both CD86 and CD206, but further studies are needed to investigate their origins. Together with the phenotypic alterations observed in CD11c^+^CD11b^+^ macrophages, it can be suggested that the MSCs stimulate phenotype shifts among macrophages towards a healthy status.

Among CD11c^+^CD11b^+^ macrophages, the M2 populations were also upregulated in the disease control group, which was consistent with the trends observed in the clinical data of the asthma patients^13,18^. The administration of the Liproxstatin-1-primed hUC-MSCs resulted in a significant reduction in the M2 macrophage population, while causing no notable change in the M1 macrophage population. This could be controversial, as M2 macrophages are generally considered to play regulatory roles and are involved in wound healing^41^. However, recent studies have revealed that asthma has also been found to be associated with an upregulation of M2 macrophages^19,20^, and M2 macrophages are known to express MRC1 on their surface and secrete FGF, Transforming growth factor- β (TGF-β), and MMP, which are highly associated with the airway remodeling seen in chronic asthma patients^42–44^. *Arg1* downregulated by MSC in M2 macrophages is known to contribute to the pathogenesis of asthma by inducing cytokine secretion in asthmatic conditions^45^ Furthermore, the effect of MSCs might be related to the reduction of *Chil3* on macrophages contributing to the development of eosinophilic lung inflammation and expression of Th2 cytokines^46,47^.

It was found that M2 macrophages exhibit high Ly6C expression only under disease conditions, and Ly6C is often appreciated as a marker for pro-inflammatory and pro-fibrotic phenotypes^48^. As Ly6C^high^ monocytes have been shown to acquire both inflammatory and regulatory phenotypes depending on exposure to the milieu^49^, it can be understood that Ly6C^−^M2 macrophages of wild-type mice may be devoted to maintaining homeostasis^50^, whereas the Ly6C^+^M2 macrophages of the IL-13 TG mice may have adapted pro-inflammatory and pro-fibrotic phenotypes in response to the asthmatic environment. When the Ly6C^+^M2 macrophages of the total macrophages were back-traced, it was found that the majority were positioned within the CD11c^+^CD11b^+^ regions (data not shown). A strong correlation between the Ly6C^+^M2 macrophages and lung eosinophil counts substantiated that the population may be highly associated with T_H_2 cells, which appear to play key roles in the aggravation of asthma.

This study has some limitations. First, although we proved that MSCs can directly suppress M2 activation, the exact mechanism was not revealed in this study. Second, we have emphasized the importance of Ly6C^+^M2 macrophages in the pathogenesis of asthma, but the direct immunologic effect of Ly6C^+^M2 macrophages has not been proven experimentally. Additional experimentation will be required to address these limitations. It is also important to note that the exact mechanism or signaling pathway that Liproxstatin-1-priming uses to enhance the functionality of naïve MSCs in anti-asthmatic activity. Although we speculate that the anti-oxidant activity of Liproxstatin-1 as an inhibitor of ferroptosis, further clarification of Liproxstatin-1 regulation of MSCs functionality would be important and needs to be determined through additional experimental settings and advanced analytic tools such as single cell or bulk expression profiling.

In conclusion, this study demonstrated the anti-asthmatic effects of Liproxstatin-1-primed hUC-MSCs and spotlighted the Ly6C^+^M2 macrophage-directed explanations of the pathogenesis of asthma. The study elucidated that the altered modulation in macrophage populations resulting from the MSC treatment could partially uncover the pathogenesis of asthma and, therefore, we suggest that macrophages may be considered as an additional therapeutic target for asthma.

## Materials and methods

### Liproxstatin-1-primed human umbilical cord-derived MSCs

MSCs were primed with Liproxstatin-1 (Sigma-Aldrich, St. Louis, MO, USA) at a 10:1 ratio 24 h prior to injection.

### IL-13 TG mice

The transgenic mice were generated and kindly donated by Professor Jack A. Elias (Department of Molecular Microbiology and Immunology, Brown University, RI, USA)^42,51^.

### Preparation of the animal model and MSC injection

Seven-week-old WT and IL-13 TG C57BL/6 mice were intratracheally injected with PBS or Liproxstatin-1-primed hUC-MSCs (1 × 10^5^/50 µL), resulting in four experimental groups: a healthy control group (WT/PBS), an MSC-treated healthy group (WT/MSC), a disease control group (IL-13 TG/PBS), and an MSC-treated disease group (IL-13 TG/MSC). The mice were sacrificed four days after MSC treatment. WT or IL-13 TG C57BL/6 mice were injected intraperitoneally with a mixture of 90 mg/kg ketamine and 10 mg/kg xylazine before sacrifice, and BAL fluid and lung samples were collected for analysis.

### Collection of BAL fluid

The trachea of the mice was exposed, and the lavage was performed twice with 1 mL of phosphate buffered saline (PBS) through an incision made in the upper trachea. BAL fluid samples were cytocentrifugated and the pellets obtained were placed on slides for differential cell counting. Further preparation methods are introduced in the online method sections.

### Histological analysis

H&E, periodic acid Schiff^52^, or MT staining processes were performed by experts at the pathology core facility at the Seoul National University Hospital Biomedical Research Institute.

### Collagen assay

A Sircol Soluble Collagen Assay kit (Biocolor, Antrim, U.K.) was purchased and subsequent steps followed the protocols provided by the manufacturer without any modification.

### Quantitative reverse transcription polymerase chain reaction (RT-qPCR)

Total RNA was isolated with Trizol (Thermo Fisher Scientific, Waltham, MA, USA.) and chloroform, and reverse transcription was performed with the SensiFAST cDNA Synthesis Kit (Bioline, London, U.K.) according to the manufacturer’s instructions. RT-qPCR was performed using the SensiFAST SYBR No-ROX Kit (Bioline, London, U.K.) according to the manufacturer’s instructions. Each gene was normalized to the expression level of the housekeeping gene *Gapdh*, *Hprt1* and the relative gene expression was calculated using the − ΔΔCt method. The primers used in this study are listed in Supplemental Table I.

### Flow cytometry

Minced lung tissues were incubated in 5 mL of RPMI1640 with 10% Type IV collagenase (Worthington Biochemical Corporation, Lakewood, NJ, USA) at 37 °C for 90 min and sorted through a sterile cell strainer for single cell preparation. Cells were blocked with Fc receptor binding inhibitor antibody at room temperature for 5 min, and then incubated with fluorochrome-labelled antibodies against cell surface markers for 30 min at 4 °C. All antibodies were purchased from BioLegend (San Diego, CA, USA). Flow cytometry was performed with the BD LSRFortessa X-20 (BD Biosciences, San Jose, CA, USA) and analyzed using FlowJo v10.6 software (BD Biosciences, San Jose, CA, USA; https://www.flowjo.com).

### Co-culture of MSCs with human peripheral blood mononuclear cell

Heparinized blood samples from house dust mite-sensitized allergic rhinitis patients were kindly donated for research purposes only. Samples were prepared as previously reported^53^. The experiment with human samples followed the guidelines of the Institutional Review Board of Seoul National University. 1 × 10^5^ PBMCs and 10 ng/mL of house dust mite (HDM) extracts (*Dermatophagoides pteronyssinus* (*Derp*-1); Stallergenes Greer, Cambridge, MA, USA) were seeded in a well plate coated with 0.5 μg/mL of CD3 and 1 μg/mL of CD28 for 24 h at 4 °C. After another 24 h incubation at 37 °C, Liproxstatin-1-primed hUC-MSCs were added at a 1:1 ratio. Activated PBMCs were co-cultured with MSCs for 48 h at 37 °C and harvested for quantitative real-time polymerase chain reaction (RT-qPCR).

### Culture of AMJ2-C11

1 × 10^5^ cells of alveolar macrophage cell line (AMJ2-C11; ATCC, Manassas, VA, USA) were seeded in a well plate. After 6 h, the cells were treated either with PBS or 20 ng/mL of recombinant IL-13 (BioLegend, San Diego, CA, USA). Naïve or Liproxstatin-1 primed-MSCs were added 12 h later. The cells were harvested after another 24 h.

### Culture of ex vivo murine alveolar macrophages and bone marrow-derived macrophages

1 × 10^5^ of ex vivo murine alveolar macrophages obtained from WT C57BL/6 mice were co-cultured with hUC-MSCs in a trans-well with a pore density of 0.4 μm (Corning, Corning, NY, USA). Six hours after seeding hUC-MSCs in the top chambers, cells in the bottom chambers were treated either with PBS or 20 ng/mL of recombinant IL-13 (BioLegend, San Diego, CA, USA). Cells were harvested 24 h after treatment for evaluation.

1 × 10^5^ of ex vivo bone marrow cells obtained from the WT C57BL/6 mouse were seeded and stimulated by 25 ng/mL of macrophage colony-stimulating factor (M-CSF) with or without 1 × 10^4^ cell of hUC-MSCs for 5 days. Cells were treated either with PBS or 20 ng/mL of recombinant IL-13 (BioLegend, San Diego, CA, USA). Cells were harvested after another 24 h.

### Statistical analysis

All statistical and graphic data were presented with Kruskal–Wallis analysis using GraphPad Prism 7 (GraphPad Software, San Diego, CA, USA, https://www.graphpad.com) unless stated otherwise. A p value less than 0.05 was considered statistically significant.

### Ethical approval

All hUC-MSCs used in this study were cultured, prepared, and primed by Professor In-kyu Kim's laboratory (College of Biomedicine, Seoul National University, Seoul, Korea) with the approval of the Seoul National University Hospital’s Institutional Review Board (SNUH IRB No. 1708-083-878). All the experiment involving human subjects was also carried out by the approval of the Seoul National University Hospital’s Institutional Review Board (SNUH IRB No. 2006-142-113) in accordance with the declaration of Helsinki and blood samples were collected after obtaining written informed consent from the patients. The experiments were approved by the Institutional Animal Care and Use Committee (IACUC) of the Institute of Laboratory Animal Resources at Seoul National University (IACUC No. SNU-200525-1-1). Less than Five mice were housed in each cage under standard conditions of temperature and humidity, according to the guidelines of Biomedical Center for Animal Resource Development at the Seoul National University in compliance with ARRIVE guideline and regulations.

## Supplementary Information

Supplementary Information.
