# Supplementary material for: Mesenchymal stem cells exert their anti-asthmatic effects through macrophage modulation in a murine chronic asthma model
Source: Sci Rep. 2022 Jun 13;12:9811. doi: 10.1038/s41598-022-14027-x (PMC9192777; doi:10.1038/s41598-022-14027-x)
Supplement: Supplementary file 1 — Supplementary Information. [file 41598_2022_14027_MOESM1_ESM.pdf]

Mesenchymal Stem Cells Exert their Anti-Asthmatic Effects Through Macrophage Modulation in a Murine Chronic Asthma Model

Anti-Asthmatic Effects of Mesenchymal Stem Cells

Ruth Lee Kim[1,2*], Ji-Young Bang[1,2*], Jeonghyeon Kim[1,2], Yosep Mo[1,2], Yujin Kim[1,2], Chun-Geun Lee[3], Jack A. Elias[3], Hye Young Kim[1,5], Hye-Ryun Kang[1,2,4#]

[1]Institute of Allergy and Clinical Immunology, Seoul National University Medical Research Center, Seoul National University College of Medicine, Seoul, Korea [2]Graduate School of Translational Medicine, Korea Seoul National University College of Medicine, Seoul, Korea [3]Brown University, 6752, Molecular Microbiology and Immunology, Providence, Rhode Island, United States [4]Department of Internal Medicine, Seoul National University College of Medicine, Seoul, Korea [5]Laboratory of Mucosal Immunology in Department of Biomedical Sciences, Seoul National University College of Medicine, Seoul, Korea

Supplemental Figure I.

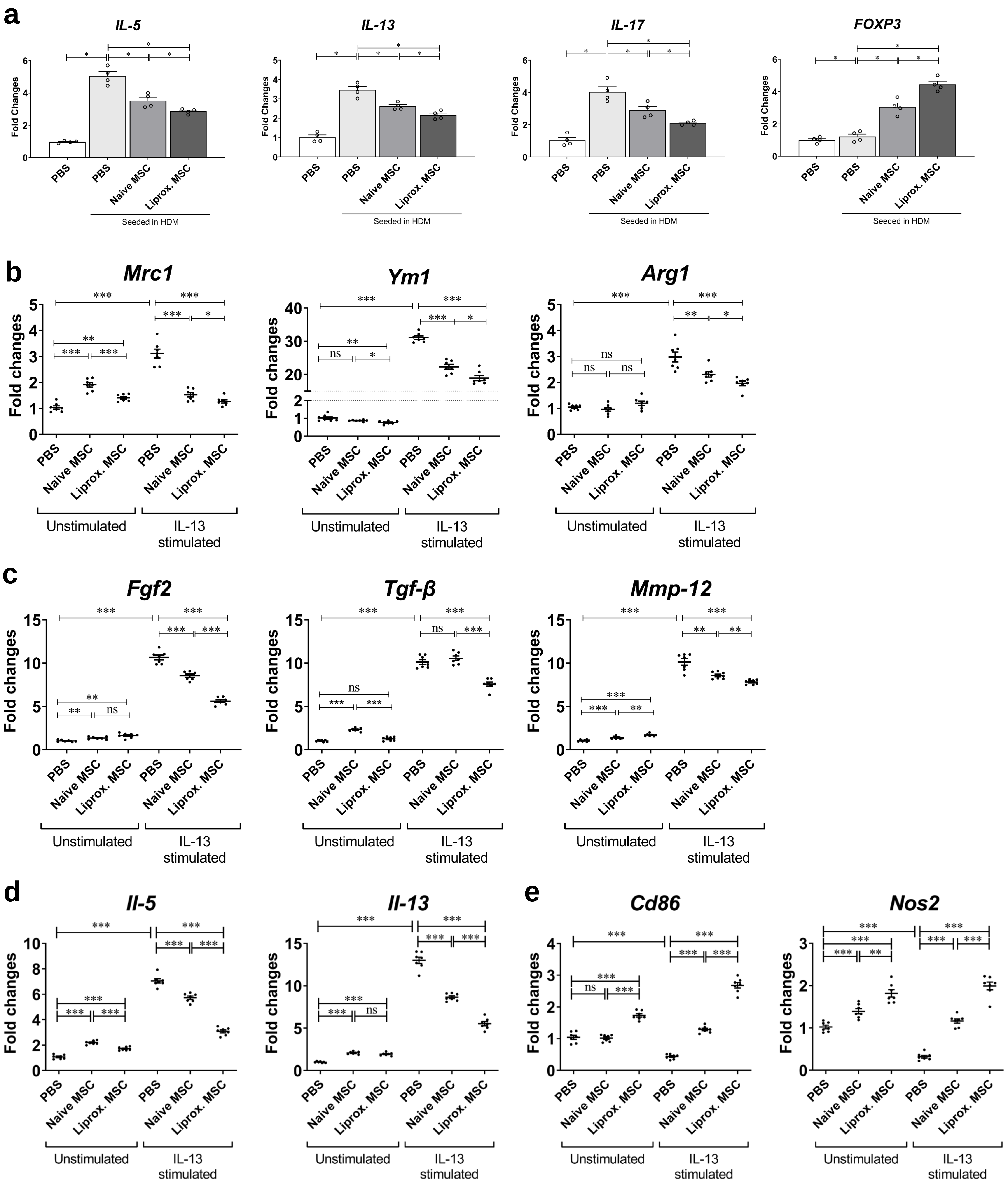

FIGURE S1. Effects of naïve and Liproxstatin-1-primed hUC-MSCs on human PBMCs or murine alveolar macrophages.

(a) Human PBMCs obtained from HDM-sensitized patients were subjected to RT-qPCR to evaluate the expression of IL-5, IL-13, IL-17, and FOXP3, the representative markers of Th2, Th17, and regulatory T cells. (b-e) Evaluation of mRNA expression of macrophages associated genes of M2 activation (b), airway remodeling (c), type 2 cytokines (d), and M1 activation (e). Each value in these panels is from a different individual and the mean±SEM is illustrated. All results are representative of at least three independent experiments. *p < 0.05, **p < 0.01, *** p < 0.001, ns, not significant. (by Kruskal-Wallis test using GraphPad Prism 7, https://www.graphpad.com) PBMC, Peripheral blood mononuclear cell; Fgf2, fibrosis growth factor 2; Tgf-β, transforming growth factor-beta; Mmp-12, matrix metallopeptidase-12; IL, Interleukin.

Supplemental Figure II.

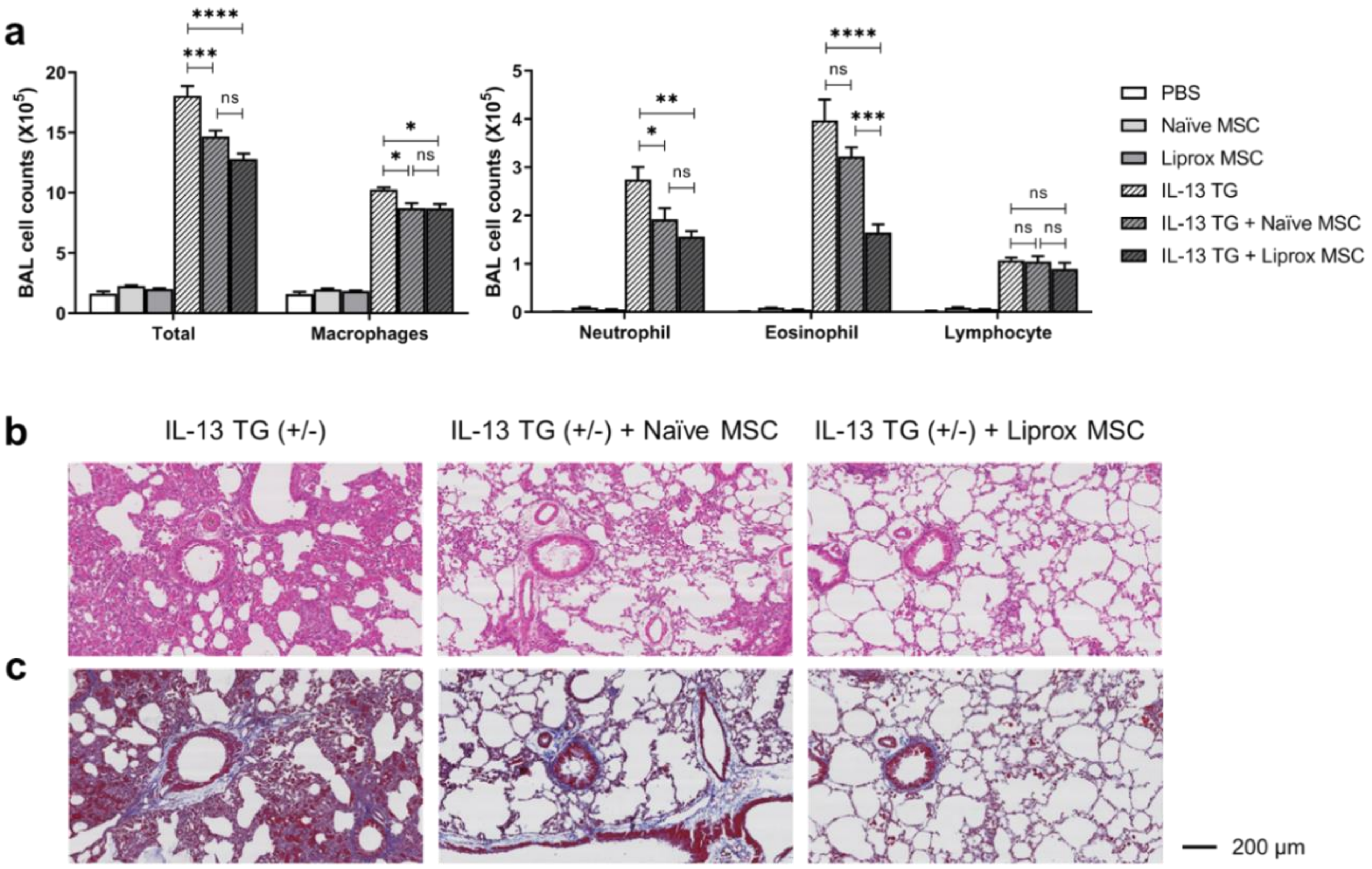

FIGURE S2 Different effects of Naïve MSC and Liproxstatin-1-primed MSC on IL-13 TG mice.

(a) shows the result of total cell counts and differential counts of macrophages, neutrophils, eosinophils, and lymphocytes in BAL fluid. **(b-c)** shows a histological comparison between the groups. H&E **(b)** and PAS **(c)** stains were used. One-way ANOVA and Tukey's analysis were used and error bars represent SEM. *p<0.05, **p<0.01, ***p<0.001, ***p<0.0001, ns stands for not significantly different.

Supplemental Figure III.

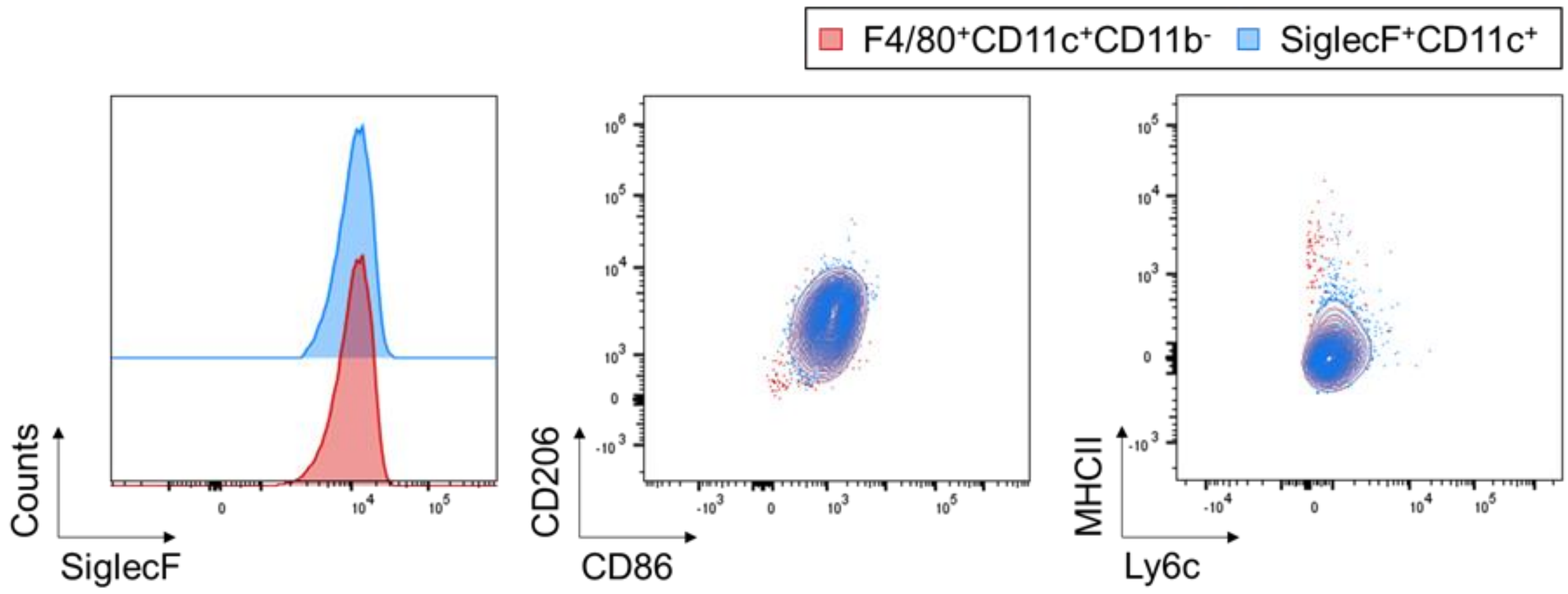

FIGURE S3 Comparison of the expression of CD206, CD86, MHCII, Ly6c, and SiglecF on SiglecF$^+$CD11c$^+$ and F4/80$^+$CD11c$^+$CD11b$^-$ alveolar macrophages assessed by flow cytometry

Supplemental Figure IV.

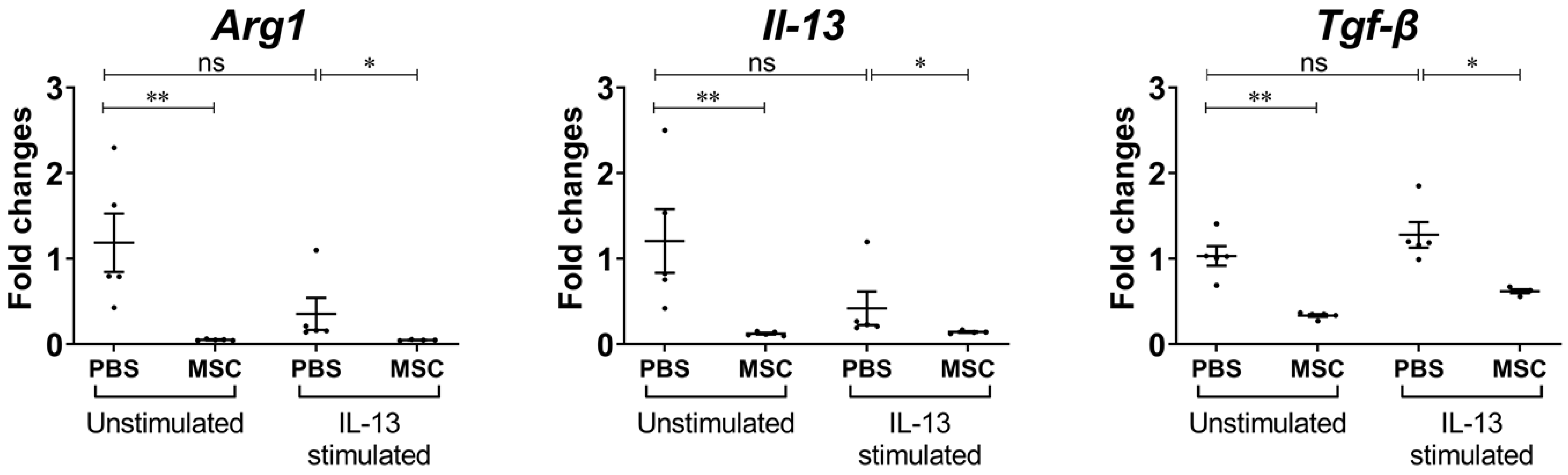

FIGURE S4 Effects of Liproxstatin-1-primed hUC-MSCs on murine macrophages.

Supplemental Table I. The primer sequences for RT-qPCR

Gene	Forward	Reverse
**Human**		
IL-5	5'-AGC TGC CTA CGT GTA TGC CA-3'	5'-GCA GTG CCA AGG TCT CTT TCA-3'
IL-13	5'-TGA GGA GCT GGT CAA CAT CA-3'	5'-CAG GTT GAT GCT CCA TAC CAT-3'
IL-17	5'-GGA CTG TGA TGG TCA ACC TGA-3'	5'-TCA TGT GGT AGT CCA CGT TCC-3'
FOXP3	5'-TCA TCC GCT GGG CCA TCC TG-3'	5'-GTG GAA ACC TCA CTT CTT GGT C-3'
GAPDH	5'-TGT AGA CCA TGT AGT TGA GGT CA-3'	5'-AGG TCG GTG TGA ACG GAT TTG-3'
**Mouse**		
Mrc1	5'-CAA GGA AGG TTG GCA TTT GT-3'	5'-CCT TTC AGT CCT TTG CAA GT-3'
Ym1	5'-GGG CAT ACC TTT ATC CTG AG-3'	5'-CCA CTG AAG TCA TCC ATG TC-3'
Arg1	5'-CTC CAA GCC AAA GTC CTT AGA G-3'	5'-AGG AGC TGT CAT TAG GGA CAT C-3'
Fgf1	5'-GGG AGA TCA CAA CCT TCG CA-3'	5'-CTG TCC CTT GTC CCA TCC AC-3'
Fgf2	5'-GAA ACA CTC TTC TGT AAC ACA CTT-3'	5'-GTC AAA CTA CAA CTC CAA GCA G-3'
Fn1	5'-CAC GGG AGC CTC GAA GAG-3'	5'-ACA ACC GGG CTT GCT TTG-3'
Tgf-β	5'-AGG AGA CGG AAT ACA GGG CT-3'	5'-CCA CGT AGT AGA CGA TGG GC-3'
Mmp-9	5'-AAC CTC CAA CCT CAC GGA CA-3'	5'-TCA TCG ATC ATG TCT CGC GG-3'
Mmp-12	5'-TCA GTC CCT CTA TGG AGC CC-3'	5'-CAC AGA TGC AGA GAA GCC CA-3'
Muc5ac	5'-GGA ACT GTG GGG ACA GCT CTT-3'	5'-GTC ACA TTC CTC AGC GAG GTC-3'
Nos2	5'-ACA TCG ACC CGT CCA CAG TAT-3'	5'-CAG AGG GGT AGG CTT GTC TC-3'
Chil3	5'-AGA AGG GAG TTT CAA ACC TGG T-3'	5'-GTC TTG CTC ATG TGT GTA AGT GA-3'
Hprt1	5'-CTG GTG AAA AGG ACC TCT CGA AG-3'	5'-CCA GTT TCA CTA ATG ACA CAA ACG-3'
Gapdh	5'-GGA GTC AAC GGA TTT GGT CGT A-3'	5'-CAA CAA TAT CCA CTT TAC CAG AGT TA-3'
